# Chito-oligosaccharides and macrophages have synergistic effects on improving ovarian stem cells function by regulating inflammatory factors

**DOI:** 10.1186/s13048-023-01143-z

**Published:** 2023-04-14

**Authors:** K. Zheng, Wenli Hong, Haifeng Ye, Ziqiong Zhou, Shuyi Ling, Yuan Li, Yuqing Dai, Zhisheng Zhong, Ziwei Yang, Yuehui Zheng

**Affiliations:** 1 Reproductive Health Department, Shenzhen Traditional Chinese Medicine Hospital, the Fourth Clinical Medical College of Guangzhou University of Traditional Chinese Medicine, Shenzhen, China; 2 Shenzhen University Health Science Center, Shenzhen, China; 3 Institute of Regenerative Biology and Medicine, Helmholtz Zentrum München, München, Germany

**Keywords:** Ovarian germline stem cells, Ovarian aging, Chitosan oligosaccharides, Macrophages

## Abstract

**Background:**

Chronic low-grade inflammation and ovarian germline stem cells (OGSCs) aging are important reasons for the decline of ovarian reserve function, resulting in ovarian aging and infertility. Regulation of chronic inflammation is expected to promote the proliferation and differentiation of OGSCs, which will become a key means for maintaining and remodeling ovarian function. Our previous study demonstrated that Chitosan Oligosaccharides (Cos) promoted the OGSCs proliferation and remodelled the ovarian function through improving the secretion of immune related factors,but the mechanism remains unclear, and the role of macrophages, the important source of various inflammatory mediators in the ovary needs to be further studied. In this study, we used the method of macrophages and OGSCs co-culture to observe the effect and mechanism of Cos on OGSCs, and explore what contribution macrophages give during this process. Our finding provides new drug treatment options and methods for the prevention and treatment of premature ovarian failure and infertility.

**Methods:**

We used the method of macrophages and OGSCs co-culture to observe the effect and mechanism of Cos on OGSCs, and explore the important contribution of macrophages in it. The immunohistochemical staining was used to locate the OGSCs in the mouse ovary. Immunofluorescent staining, RT-qPCR and ALP staining were used to identify the OGSCs. CCK-8 and western blot were used to evaluate the OGSCs proliferation. β-galactosidase(SA-β-Gal) staining and western blot were used to detect the changing of cyclin-dependent kinase inhibitor 1A(P21), P53, Recombinant Sirtuin 1(SIRT1) and Recombinant Sirtuin 3(SIRT3). The levels of immune factors IL-2, IL-10, TNF-α and TGF-β were explored by using Western blot and ELISA.

**Results:**

We found that Cos promoted OGSCs proliferation in a dose-and time-dependent manner, accompanied by IL-2, TNF-α increase and IL-10, TGF-β decrease. Mouse monocyte-macrophages Leukemia cells(RAW) can also produce the same effect as Cos. When combined with Cos, it can enhance the proliferative effect of Cos in OGSCs, and further increase IL-2, TNF-α and further decrease IL-10, TGF-β. The macrophages can enhance the proliferative effect of Cos in OGSCs is also associated with the further increase in IL-2, TNF-α and the further decrease in IL-10, TGF-β. In this study, we determined that the anti-aging genes SIRT-1 and SIRT-3 protein levels were increased by Cos and RAW respectively, whereas the senescence-associated SA-β-Gal and aging genes P21 and P53 were decreased. Cos and RAW had a protective effect on OGSCs delaying aging. Furthermore, RAW can further decrease the SA-β-Gal and aging genes P21 and P53 by Cos, and further increase SIRT1 and SIRT3 protein levels in OGSCs by Cos.

**Conclusion:**

In conclusion, Cos and macrophages have synergistic effects on improving OGSCs function and delaying ovarian aging by regulating inflammatory factors.

## Introduction

The view that most female mammals incapacitated oocyte production at birth has been debated in recent years by the finding that postnatal ovaries of mouse exist mitotically active germ cells [1]. Recently, multiple research teams including us, have used a variety of methods to prove the existence of OGSCs, such as stem cell culture and tissue/stem cell transplantation in vitro, genetic modification and in vivo lineage tracing [2]. OGSCs have been confirmed existing in ovary after birth in a variety of mammals, such as mice, sheep, pigs and humans (including menopausal women), and it has been observed that OGSCs can self-renew, clone expansion and have the ability to directionally differentiate into oocytes, continuously renew follicular pool, and restore the ability to produce offspring in infertile model animals after transplantation [3–7]. Like a normal ovary, the ovaries are filled with follicles and secrete hormones. Through single-cell sequencing, Wu found that the ovarian organoids contained several cell groups, such as germ cells, granulosa cells and follicular membrane. Follicles removed from ovarian organoids have ability to develop into mature oocytes in vitro. Mature oocytes generate offspring by in vitro fertilization [8]. These indicate that the abnormal function and decreased number of OGSCs are the decisive cause of human ovarian aging, and reactivating OGSCs is an important means to reshape and protect ovarian function. However, how to activate endogenous OGSCs with low immunogenicity, controllable analysis, and no ethical problems and achieve in situ remodeling of ovarian function remains to be explored.

Accumulating researches suggest hostile OGSCs niche is the key link to ovarian failure. Researchers think the damaged stem cell niche can cause stem cell telomere shortening, DNA damage accumulation, abnormal gene expression, abnormal cell signaling, further affect stem cell self-renewal, senesence, skewed differentiation and compromised regeneration [9]. Niche is the microenvironment for stem cells inhabit. The gerenal niche consist of niche cells, extracellular matrix, granulocytes et al., but OGSCs niche composition and function are far less clear. Bukovsky found that OGSCs niche shaped in early embryonic development period, which are composited of nonspecific ovarian monocyte-derived cells(MDCs), vascular endothelial cells, and T cells, whereas niche of adult OGSCs are formed of primary CD14 + MDCs, activated HLA-DR + MDCs and T cells [10]. We think the immune cells in the niche, such as macrophages, can secrete many cytokines, including IL-10 and TNF-α, which help to slow the OGSCs aging [11]. We hypothesized that regulating the function of Mφ residing in ovary is the key link to activate endogenous OGSCs.

OGSCs are bi-potent stem cells from ovarian surface epithelium, which exists a role in producing new stem cell by symmetrical division or differentiate into oocytes and primordial granulosa cells by asymmetric division [12, 13]. OGSCs are a type of adult stem cells. More and more experimental studies have proposed that the decline in the number and abnormal function of tissue adult stem cells are an important cause of body aging [14, 15]. Therefore, some scholars believe that ovarian aging is caused by the loss of OGSCs, and the expansion/refilling of OGSCs can play a role in preventing and treating ovarian aging [16, 17]. Therefore, the aging of OGSCs may be the root cause of ovarian aging! Mφ are the largest number of innate immune cell in the ovary. They generate various inflammatory mediators and play a central role in maintaining tissue homeostasis [18]. Macrophages, neutrophils, and NK cells are located in the hypothalamic–pituitary–ovarian axis tissues and secrete the cytokines (IL-6, TGF-β, TNF-α, and IL-2 etc.) which affect hormonogenesis and hormone secretion [19]. Zhang obseved a significant increase in monocyte recruitment and macrophage alternative, activation (M2) in the aged ovaries compare to macrophage populations and polarization in young [20]. Nowadays, much research has found an increasingly close relationship between deficient immune function, inflammatory, and aging-related pathologies [9]. The character and development of aging-associated changes in immune systems and the effect of immunity on homeostasis are significant for exploring the process of aging. However, current research on the relationship between macrophage and OGSCs aging and how to regulate the function of Mφ is essentially rare.

Chitosan oligosacchrides (Cos), which is mainly derived from crustaceans, insects, bacteria, algae, and higher plant cytoderm, is the second largest biological resource on the Earth [21]. To date, Cos is the only polysaccharide in nature with positive charge, alkaline, intrinsic water-soluble characteristics, and has no toxic side effects but good biocompatibility. Cos applies in food, medicine, chemicals, environmental protection, cosmetics, and agriculture fields and is known as the "sixth life element" (top five: glucide, vitamins, protein, lipid, minerals) by the biomedical community ( [22]: induced by The National Health and Family Planning Commission, 2014). Our previous study demonstrated that intragastric administration of Cos significantly increased the ovarian index, decreased the rate of follicular atresia, increased the secretion of E_2_ and AMH, and increased the expression of IL-2 and TNF-α in the ovaries; in vitro experiment showed Cos alleviate H_2_O_2_-stimulated granulosa cell damage, when Cos and OGSCs were co-cultured, resulting in significantly proliferation of OGSCs and accompanied by an increase in cytokines of IL-2 and TNF-α. These indicated that Cos promoted the OGSCs proliferation and remodelled the ovarian function through steadying the ovarian microenvironment and stimulating the production of immune-related factors [23, 24]. However, the mechanism by which Cos promotes OGSCs proliferation remains unclear, and the role of Mφ in this effect needs to be further studied. In this study, we used the method of macrophage and OGSCs co-culture to observe the effect and mechanism of Cos on OGSC, and explore what contribution macrophage gives during this process. Our finding provides new drug treatment options and methods for the prevention and treatment of premature ovarian failure and infertility.

## Materials and methods

### The isolation of mouse OGSCs

Kunming mice were purchaesd from the Medical Animal Center of Nanchang University. The animals for this study had been obtained the Nanchang University animal ethnic approval. Ovaries from 3-to 5-day-old neonatal mice were excised in aseptic environment and rinsed with D-Hanks liquid individually. The OGSCs were isolated by the modified two-step enzymatic method [24, 25]. The entire ovaries were placed in a 15 ml centrifuge tube containing 5 ml D-Hanks liquid and 1 mg/ml of collagenase type IV (C4-28-100MG, 1 mg/ml, Sigma, USA) and shook gently at 37 °C for 30 min in water bath until the ovarian tissue were digested into flocculent state. Then centrifuged at 1500 rpm for 5 min and the supernatant was discarded. After that, added 5 ml trypsin–EDTA (Sigma, USA) to the centrifuge tube and shook gently at 37 °C for 2 min in water bath. Added DMEM medium with 10% FBS to end digestion, then centrifuged in a 4 °C centrifuge at 1500 rpm for 5 min and discarded the supernatant. The precipitate was resuspended with 1 ml D-Hanks liquid and centrifuged again. Then removed supernatant and plated in STO feeder cell paved 48-well plate after resuspended with 200 µl OGSCs nutrient fluid (Table 1). The cultures were incubated at 37 °C with 5% CO_2_.

**Table 1:** The Mixed Medium of OGSCs

Name (brand)	Volume	Concentration
β-mercaptoethanol (Sigma)	0.07 μl	0.1 mM
epidermal growth factor (Sigma)	1 μl	10 ng/ml
basic fibroblast growth factor (Sigma)	1 μl	1 ng/ml
mouse glial cell line-derived neurotrophic factor (Sigma)	1 μl	40 ng/ml
mouse leukemia inhibitory factor (Sigma)	2 μl	20 ng/m
non-essential amino acids (Gibco)	100 μl	1 mM
L-glutamine (Sigma)	100 μl	2 mM
sodium pyruvate (Gibco)	100 μl	1 mM
penicillin and streptomycin (Solarbio)	100 μl	100 ×
FBS (Gibco)	1 ml	–
minimum essential medium alpha (MEM-α; Invitrogen)	Up to 10 ml	–

### Processing of macrophage ( RAW) feeder cell layer

The frozen RAW cells were quickly removed from the liquid nitrogen and placed in a water bath at 37 °C. The cell suspension was added to a 15 ml centrifuge tube, centrifuged at 300 g for 5 min. Then, put into the dishes and cultured at 37 °C in 5% CO_2_. When cells entered into the logarithmic phase, removed the medium and added 10 u g/ml mitomycin-C, and incubated for 2 h. Cells were collected and centrifuged, then spread them to a 48-well plate in the appropriate concentration, further culturing at 37 °C in 5% CO_2_. The RAW cells was co-cultured with OGSCs via transwell manner.

### The identification of mouse ovarian germline stem cells

#### Immunofluorescent staining

Cells were washed twice with PBS on 96-well plate, fixed in 4% polyoxymethylene for 20 min at room temperature, permeabilized with 0.5% Triton X-100 for 15 min and then blocked with 1% BSA -TBST for 30 min. Cells were incubated with primary antibodies (OCT4, ab184665, Abcam, USA; MVH, ab270534, Abcam, USA) overnight at 4 °C. The fluorescence-labeled secondary antibody (A-11004, A32731, Thermo, USA) was incubated for 1 h at room temperature. Nuclei were stained by DAPI (D1306, Invitrogen, USA) for 10 min. Images were examined with an Olympus microscope(IX73, Olympus, Japan).

#### Reverse transcription- PCR

Total RNA extraction from cell plate were using the Trizol method(Invitrogen, Germany), and the measured optical density was detected by 2% agarose gel electrophoresis(Bio-Rad, USA) after RNA concentration and purity detection(IMPLEN, Germany). The primers used in the present study are listed in Table 2.

**Table 2:** The Sequences of Primers Used for Reverse Transcription- PCR

Gene	Forward primer (5'-3')	Reverse primer (5'-3')
GAPDH	AACGGATTTGGCCGTATTGG	CATTCTCGGCCTTGACTGTG
MVH(DDX4)	GTGTATTATTGTAGCACCAACTCG	CACCCTTGTACTATCTGTCGAACT
Fragilis	CTGGTCCCTGTTCAATACACTCTT	CAGTCACATCACCCACCATCTT
OCT-4(Pou5f1)	AGCTGCTGAAGCAGAAGAGG	GGTTCTCATTGTTGTCGGCT
Stella	CCCAATGAAGGACCCTGAAAC	AATGGCTCACTGTCCCGTTCA
Dazl	GTTAGGATGGATGAAACCGAAAT	ATGCCTGAACATACTGAGTGATA
c-Kit	CGCCTGCCGAAATGTATG	TCAGCGTCCCAGCAAGTC
ZP3	GAGCTTTTCGGCATTTCAAG	AGCTTATCGGGGATCTGGTT
Scp3	GAGCCGCTGAGCAAACATCTA	ATATCCAGTTCCCACTGCTGC
Figla	CCAAAGAGCGTGAACGGATAA	TCTTCCAGAACACAGCCGAGT

#### Alkaline phosphatase (ALP) staining

Cells were seeded in a 48-well plate and stained on 3^rd^ day after isolation. Cells were washed twice with PBS and stained according to the manufacturer's protocol(C3206, Beyotime, China). Staining was observed with a microscope (IX73, Olympus, Japan).

### Western blot

Western blot was performed according to the manufacturer's specifications. The primary antibodies used were MVH (ab27591, Abcam, USA), OCT4 (ab18976, Abcam, USA), TNF-α (EPR22598-212, Proteintech, USA), IL-2 (26,156–1-AP, Proteintech, USA), IL-10 (Abcam, ab189392), TGF-β(ab179695, Abcam, USA). SIRT1(ab12193, Abcam, USA), SIRT3 (ab189860, Abcam, USA), p21(28,248–1-AP, Proteintech, USA), p53 (10,442–1-AP, Proteintech, USA), GAPDH (ab181602, Abcam, USA). All the HRP secondary antibodies were obtained from Affinity Biosciences. After accordingly antibody incubation and washing with TBST, PVDF membranes were imaged using an EasySee Western blot kit (DW101–01, TransGen, China). Images AI600 and Image J were used to scan and analyze the images.

### SA-β-gal assay

SA**-**β-gal assay was measured according to the manufacturer's specifications (C0602, Beyotime, China). OGSCs cultured in 6-well plate were washed with PBS two times, added 1 ml β‐galactosidase fixative per well for 15 min at room temperature. Then washed twice with PBS and stained in 1 ml staining solution (50ul X-Gal solution + 10 ul A solution + 10 ul B solution + 930 ul C solution) per well at 37 °C (without CO_2_). After staining 12 h, OGSCs washed triple with PBS. Cells were photographed under Olympus microscope (IX73, Olympus, Japan).

### Cell Counting Kit-8

Cell growth was assessed using a Cell Counting Kit-8 (CCK-8, APExBIO, USA) following the manufacturer's protocol at 24, 48, 72 and 96 h, followed by measuring the spectrophotometric absorbance( DeTie, China) at 450 nm was used to estimate cell proliferation.

### Enzyme-Linked Immunosorbent Assay (ELISA)

The concentration of IL-2, TNF-ɑ, IL-10 and TGF-β in the culture medium of OGSCs cultured with Cos was determined by using an ELISA kit (Westang, China).

### Immunohistochemistry

Immunohistochemistry of mice ovaries was performed according to the previous study(Hashimoto et al., 2019). The primary antibodies used were MVH (ab27591, Abcam, USA), OCT-4 (ab181557, Abcam, USA), the secondary antibodies were obtained from Thermo. Cells were photographed under Olympus microscope (IX73, Olympus, Japan).

### Statistical analysis

All measurement data which in accordance with normal distribution were represented as Mean ± S.E.M. The compare between measurement data were tested by one-way Analysis of Variance(ANOVA), *P* < 0.05 was considered to be statistically significant.

## Results

### OGSCs localization

**Fig. 1:**
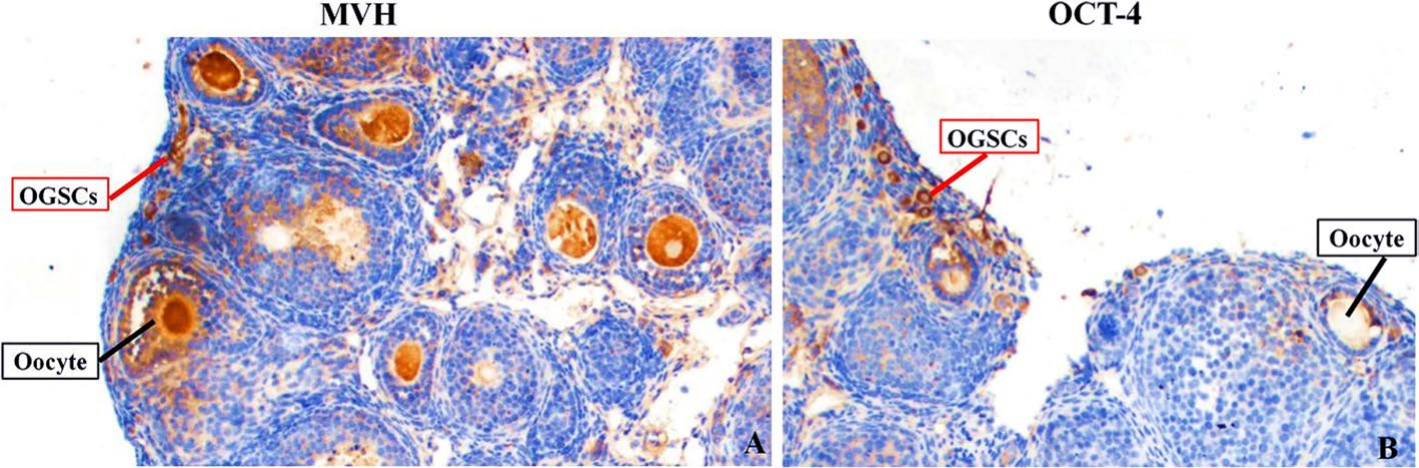
Localization of Mouse OGSCs in the Ovary. **A** Immunohistochemistry of MVH in in OGSCs cells and oocytes of all stages in ovarian cortex; **B** Immunohistochemistry of OCT-4 in OGSCs cells in ovarian cortex. Scales bar: 100 μm

Mouse vasa homologue(MVH) and octamer-binding transforming factor 4(OCT-4) are the most commonly used markers of OGSCs in mouse ovaries. Ovaries of 4-week-old mice were sectionalized by paraffin and immunohistochemical staining. As shown in Fig. 1A, MVH was positively expressed in OGSCs cells and oocytes of all stages in varian cortex. While shown in Fig. 1B, OCT-4 was only positively expressed in OGSCs cells in mouse ovarian cortex, but not in oocytes at all stages. The results showed that the mouse OGSCs was located in ovarian surface epithelium. This founding was similar to previous studies [26–29].

### OGSCs identification

**Fig. 2:**
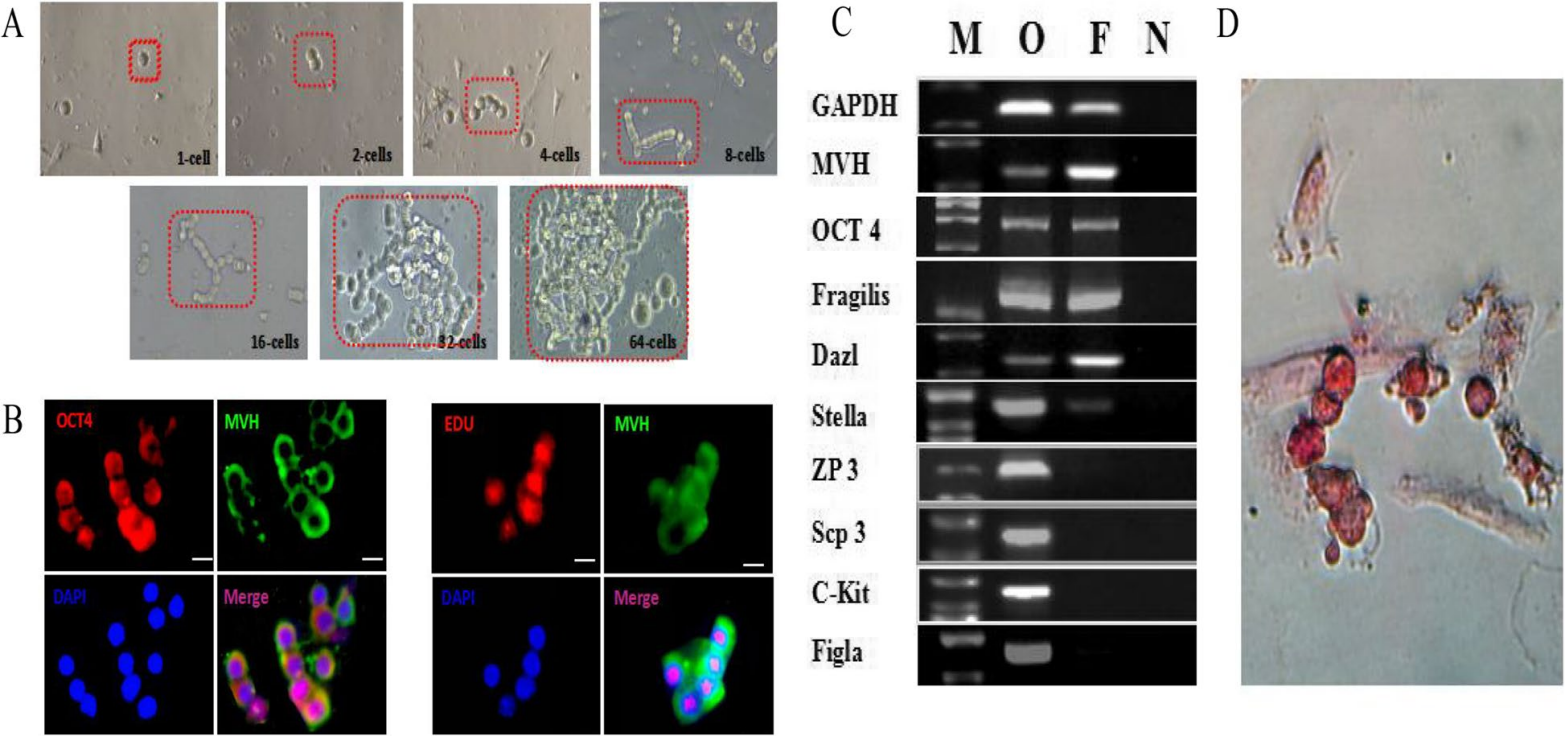
Localization of Mouse Ovarian Germline Stem Cells in the Ovary. **A** Single-cell cloning of OGSCs derived from a two-step enzymatic method; **B** Immunofluorescence of OCT-4 and EDU on OGSCs; **C** RT-qPCR identification (M: mark; O: ovary; OG: OGSCs; N: negtive)and **D** ALP staining of OGSCs. Scales bar: 10 μm

We performed identification experiments to verify the OGSCs. In Fig. 2A, isolated OGSCs were circular shape with a diameter of 15–20 μm. We observed that the cell proliferation cycle was about 1–2 d. After developed to the 4–5 th generations, they were in shape of beaded or colony-shaped, meanwhile, accompanied with a portion of cell apoptosis and differentiation into oocytes.

Immunofluorescence staining was performed using the reproductive marker gene MVH and the stem cell-specific gene OCT-4. The results showed that both marker genes were expressed in OGSCs. Simultaneously, we found the proliferation marker EDU and the germline-specific cytosolic MVH expressed in OGSCs, which proved that the OGSCs existed a certain role in proliferation (Fig. 2B.).

The level of mRNA was detected from OGSCs for 7th d, the RT-qPCR results showed that OGSCs expressed MVH, Fraglis, Dazl, OCT4, Stella, but not ZP3, Scp3, C-Kit, figla.We also found that positive for Alkaline phosphatase(ALP) staining. These results indicated that the cells isolated were preliminarily identified as OGSCs (Fig. 2C.).

### RAW enhanced the proliferation effect, anti-aging and anti-inflammation of Cos on OGSCs

**Fig. 3:**
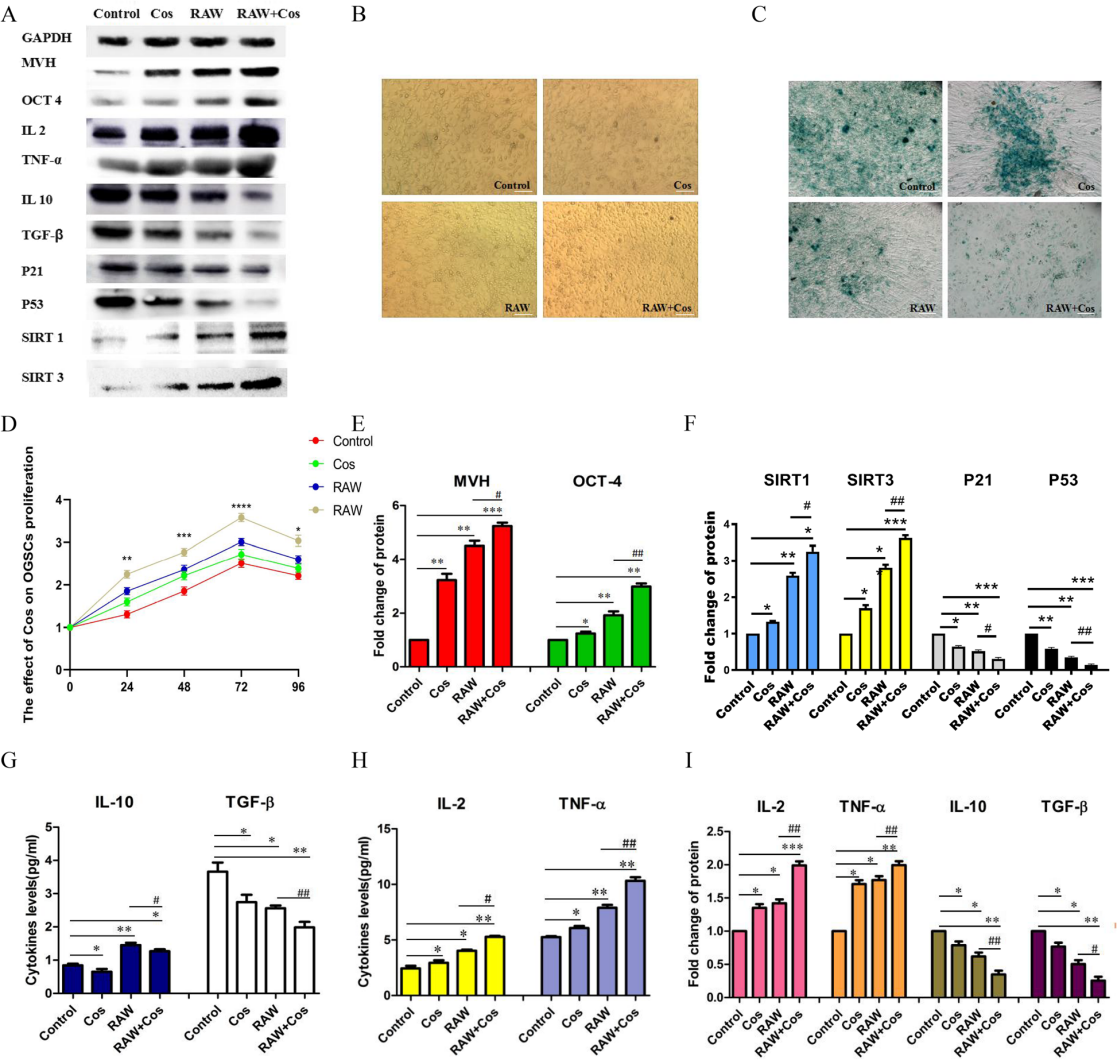
RAW Enhanced the Proliferation Effect, Anti-aging and Anti-inflammation of Cos on OGSCs. **A** The relative protein levels; **B** The morphology of OGSCs at 72 h; **C** The SA-β-Gal expression of OGSCs. **D** The proliferation vitality curves of OGSCs; **D**, **E**, **F** and **I** The statistics histogram of western blotting were expressed as band density normalized versus GAPDH; **G** and **H** The levels of immune factors in supernatant, *p < 0.05, **p < 0.01, ***p < 0.001, * compared with the Control group; #*p* < 0.05, ##*p* < 0.01, ###*p* < 0.001, # compared with the RAW group. Scale bar: 50 μm

The cell proliferation of OGSCs was measured by CCK-8. According to Fig. 3D, in the Cos group and RAW group, cell proliferation increase compared to the control group (*P* < 0.05 at 72 h). In the RAW + Cos group, the proliferation vitality are higher than the other three groups at 24 h, 48 h, 72 h and 96 h (P < 0.05 at 24 h;*P* < 0.01 at 48 h; *P* < 0.001 at 72 h). Figure 3B is the OGSCs morphology of the four groups at 72 h, we found that both RAW and Cos increased cell density,and the cell density was highest in RAW + Cos group. Quantification of reproductive marker gene MVH and the stem cell-specific gene OCT-4 in OGSCs experiment showed that both Cos and RAW protein increased. While in the RAW + Cos co-treatment group, protein level of MVH and OCT-4 production significantly increased compared to COS group or RAW group alone(Fig. 3A, E).

SA-β-Gal staining showed SA-β-Gal expression (blue cells) decreased after the addition of Cos and RAW group alone, meanwhile SA-β-Gal significantly decreased when co-cultured with Cos + RAW (Fig. 3C.). Western blot were used for quantitative analysis of aging-related genes, including aging genes P21, P53 and anti-aging genes SIRT-1, SIRT-3 in OGSCs. The results indicated that Cos dramatically inhibited the expression of P21, P53 and increased the level of SIRT-1 and SIRT-3,when OGSCs co-cultured with Cos + RAW. It has synergistic inhibitory effect on the expression of aging genes and synergistic promoting effect on the expression of anti- aging (Fig. 3A, F).

We used western blot and ELISA to detect the levels of immune factors IL-2, IL-10, TNF-α and TGF-β. Supernatants were collected from four groups and showed that pro-inflammatory factors IL-2 and TNF-α increased in Cos and RAW group alone, and were highest in the RAW + Cos group. Meanwhile, anti-inflammatory factors IL-10 and TGF-β decreased in Cos or RAW group alone, and were lowest in the RAW + Cos group (Fig. 3G, H). The western blot results were consistent with that of ELISA (Fig. 3A, I).

## Discussion

Infertility affects approximately 48.5 million world-wide females, and it is gradually increasing as the trend of first pregnancy age delay continues in both developed and developing countries [30]. Ovarian aging is the major cause of infertility and includes physiological factors such as age and pathological factors such as radiotherapy, chemotherapy, and surgery. The first aging organ in the human body is the thymus. Concurrently, ovarian aging occurs, which begins with the embryo's oocyte death at 20 weeks of gestation [31]. Modern medical professionals generally believe that the decline in the number of primordial follicle caused by various factors is an important cause of physiological and pathological ovarian aging resulting in infertility and menopause. It is also the main cause of infertility and failure in assisted reproductive technology (ART) [32]. Notably, ovarian aging is associated with whole body pathological states such as diabetes, cardiovascular and cerebrovascular diseases, cancer, and other chronic diseases. In view of this, some scholars compare ovarian aging to pacemaker of female body aging [33]. Current studies have shown that chronic low-grade inflammation and ovarian stem cells aging are important reasons for the decline of ovarian reserve function, resulting in ovarian aging and infertility. Chronic low-grade inflammation exists in pathological conditions such as premature ovarian failure, physiological aging of the ovaries, and polycystic ovary syndrome, local inflammation is disruptive to ovarian function and oocyte development [34–37]. Banerjee’s team observed that abnormal spindle organization and chromosome alignment in metaphase II (MII) mouse oocytes occurred in exposure to elevated level of pro-inflammatory cytokine IL-6, which demonstrated a direct relationship between almighty cytokine activity and the feeble meiotic competence of oocyte [38]. Moreover, obesity-induced activation of inflammatory pathways might exert negtive impacts on follicular development and oocyte quality [39]. Several mice experiments suggested that disruption of the NLR Family Pyrin Domain Containing 3 (NLRP3) inflammasome, which may be the root of serial pathologies related to aging, having a major influence on inflamm-aging, reduces inflammation, prolongs the fertile window and delays ovarian aging process [33]. Therefor, we deduce from above founding that the gradually aggravated chronic inflammation in the ovary may contribute to the low oocyte quality, and the development of pathology during ovarian aging process.

The innate immune cells play active roles in the inflammatory response. Macrophages are the largest number of innate immune cells in the ovary, which regulate follicle growth, tissue remodeling at ovulation and corpus luteum transition. As a highly diverse and plastic population of phagocytes, they are main sources of multifarious inflammatory mediator, macrophages play a extensive role in maintaining tissue homeostasis [40]. During the response to the tissue microenvironment, macrophages polarize to M1 (classically-activated) state to remove pathogens and damaged cells, or M2 (alternatively-activated) state to remodel ECM and promote tissue repair [41, 42]. However, whether macrophage can regulate the function of OGSCs is essentially barren. OGSCs are bi-potential stem cells derived from the ovarian cortex, which can produce a new stem cell by symmetrical division and primitive granulosa cells by asymmetrical division [43]. In recent years, the discovery of OGSCs and more OGSCs functions being detected have brought unprecedented expectations for female infertility treatment and improving of ovarian aging [2, 44, 45]! Nowadays, OGSCs has been discovered for more than 10 years, but they are still in the basic research stage and need further exploration. Bukovsky proposed immune system related cells and molecules participate in OGSCs asymmetrical divisions, cause OGSCs developing into oocytes and primordial granulosa cells, so as to maintain the normal function of the ovaries [46, 47]. The quantity of OGSCs were most abundant in the newborn mouse ovaries. OGSCs undergo an aging process like most of other cells, decline their capacity to divisive and increase apoptosis of developing oocytes. Also the difficulty of isolating OGSCs increases with aging [9, 48]. At present, a small number of studies by us and others have shown that appropriately improving the inflammatory and oxidative stress microenvironment for OGSCs can promote the proliferation of OGSCs and delay their aging, and also delay ovarian aging [13, 49].

Several studies reported that Cos highly improved the phagocytic function of mouse peritoneal macrophages via peritoneal Cos injection or intragastric Cos administration, leading to the results of an increase in the immune organ index such as thymus and spleen. Cos stimulated the cytokines secretion by activating macrophages, then a series of cascade reactions followed up [22, 50]. Previous studies found that macrophages existed in the tissues of the hypothalamic–pituitary–ovarian axis, and they secreted cytokines (TGF-β, TNF-α, IL-2, and INF-γ) which affected hormone generation [51]. Besides, macrophages-derived cytokines exerted a significant impact on regulating ovarian function. So we detected whether supplement with Cos and macrophages in vitro can play a protective role in OGSCs function.

**Fig. 4:**
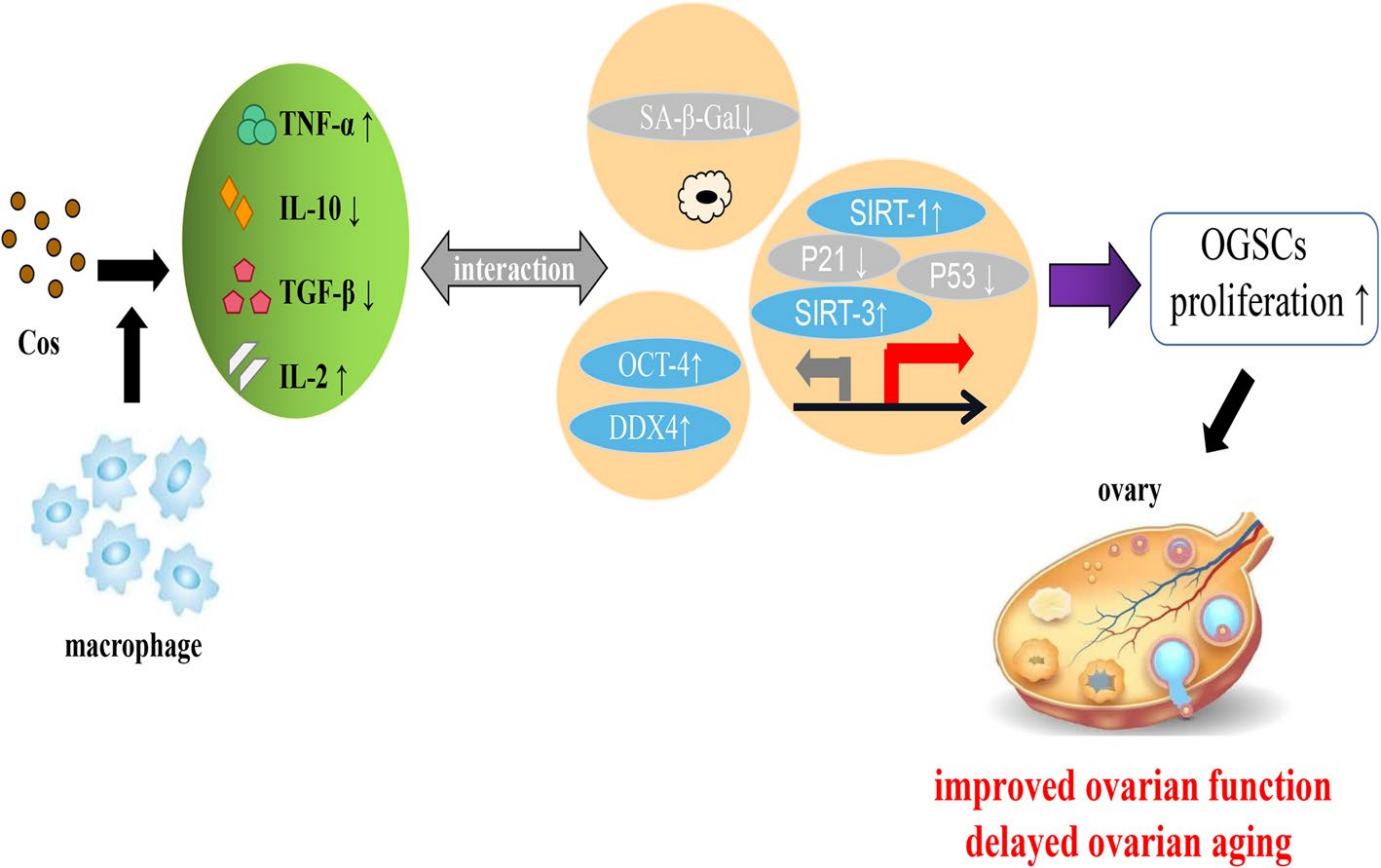
Cos Improved Ovarian Function and Delayed Ovarian Aging via Modulating Macrophage’s Function

In this study, we found that Cos promoted OGSCs proliferation in a dose-and time-dependent manner, accompanied by IL-2,TNF-α increase and IL-10, TGF-β decrease. RAW can also produce the same effect as Cos. When combined with Cos, it can enhance the proliferative effect of Cos in OGSCs, and further increase IL-2,TNF-α and further decrease IL-10, TGF-β. IL-2 is an interleukin that can regulate the activity of leukocytes in the immune system, strengthen Th0 and CTL proliferation, and also participate in antigen–antibody response, hematopoiesis, and immune surveillance of tumors [52]. TNF-α kills and restrains the growth of tumor cells, also promotes cell proliferation and differentiation. TNF-α has obvious synergistic action for proliferative effect of EGF, PDGF and insulin, and it enhances the expression of EGF receptor [53]. IL-10 is a multifunctional, multicellular-derived cytokine that revolves in cell proliferation, differentiation and response of immune, it can also suppress the secretion of TNF-α [54]. TGF-β inhibits the proliferation of immune cells and TNF-α production [55]. Inflammatory cytokines have been predominately studied in macrophages and other innate immune cells. Furthermore, the expression of NLRP3 protein and the adaptor molecule ASC, a major inflammasome on inflammaging, previously detected in the mouse oocytes and theca cells [56, 57]. In this study, we found that OGSCs is also an important source of cytokines, and Cos can significantly promote the production of pro-inflammatory cytokines in OGSCs, while inhibiting the production of anti-inflammatory cytokines. The results are consistent with Zhang's observations, he think elevated anti-inflammatory cytokines might alter extra cellular matrix (ECM) profile that resulted in extensive tissue fibrosis [58]. Therefore, Cos promoted cell proliferation of OGSCs maybe via increasing the secretion of IL-2, TNF-α, reducing the secretion of IL-10 and TGF-β. The macrophages can enhance the proliferative effect of Cos in OGSCs is also associated with further decrease IL-2,TNF-α and further increase IL-10, TGF-β (Fig. 4).

The aging of stem cells is determined by both anti-aging and aging genes, and SA-β-Gal is an important marker of cell aging. SIRT1 gene is recognized as a highest homology of Silence information regulator–related enzymes (sir2 gene family), which is involved in the regulation of mammalian germline function [59]. SIRT1 function is closely related to female reproductive diseases, such as endometriosis, polycystic ovary syndrome, and aging-related infertility, by mediating caloric homeostasis, improving mitochondrial function, and affecting chromatin recombination. The downregulation of SIRT1 expression in ovary linearly correlated with follicular physiological or pathological loss [59, 60]. SIRT1 protein is a highly conserved deacetylase dependent on NAD + , which can regulate cell proliferation or apoptosis by acetylating downstream substrates such as p53, FOXO1, NF-κB, and so on. One of the key roles of SIRT1 is the deacetylation of p53 at the C-terminal lysine-382 residue by NAD + , which reduces p53-mediated transcriptional activity, thereby reducing the expression of downstream proteins, such as p21 (cell cycle inhibitor). Deacetylation of SIRT1 can inhibit p53-regulated cell cycle arrest and apoptosis, enhance the mechanism of DNA repair, promote the maintenance of genomic integrity, and promote cell survival and proliferation. In this study, we determined that the anti-aging genes SIRT1 and SIRT3 protein levels were increased by Cos and RAW respectively, whereas the SA-β-Gal and aging genes P21 and P53 were decreased. Cos and RAW had a protective effect on OGSCs delaying aging. Furthermore, RAW can further decrease the SA-β-Gal and aging genes P21 and P53 by Cos, and further increase SIRT1 and SIRT3 protein levels in OGSCs by Cos. All the results suggested the effect of Cos improving proliferation and delaying aging in OGSCs may be via regulating inflammatory cytokines, and then activates anti-aging and inhibits aging signaling pathways. Macrophages can mediate and enhance the effect of Cos on promoting OGSCs proliferation by regulating the production of cytokines. Cos is expected to become a natural drug to maintain and reshape ovarian function!

## Concluison

In conclusion, our research demonstrates that Cos can significantly promote OGSCs proliferation and delay aging by regulating cytokines secretion and the macrophages can mediate and enhance the effect of Cos on promoting OGSCs proliferation by regulating the production of cytokines,the mechanisms are related to decreasing the SA-β-Gal and aging genes P21 and P53 levels, increasing SIRT1 and SIRT3 protein levels in OGSCs. Our study provides potential therapeutic strategies to prolong reproductive lifespan for aged women.

## Data Availability

The data used to support the findings of this study are available from the corresponding author upon request.
